# Patient Experiences Regarding Feasibility of Implementing Real-World EQ-5D Collection at an Oncology Centre in Ontario, Canada

**DOI:** 10.3390/curroncol32060308

**Published:** 2025-05-27

**Authors:** Teresa C. O. Tsui, Rebecca E. Mercer, Elena J. Zhou, Rahul K. Desai, Shreya Chatterjee, Curtis Y. L. Yeung, Eleanor M. Pullenayegum, Kelvin K. W. Chan

**Affiliations:** 1 Sunnybrook Research Institute, Toronto, ON M4N 3M5, Canada; teresa.tsui@alumni.utoronto.ca (T.C.O.T.); rebecca.mercer@sunnybrook.ca (R.E.M.); ejzhou@uwaterloo.ca (E.J.Z.); rkdesai@uwaterloo.ca (R.K.D.); shreya.chatterjee@uwaterloo.ca (S.C.); cy7yeung@uwaterloo.ca (C.Y.L.Y.); 2 Dalla Lana School of Public Health, University of Toronto, Toronto, ON M5S 1A1, Canada; eleanor.pullenayegum@sickkids.ca; 3 Canadian Centre for Applied Research in Cancer Control (ARCC), Toronto, ON M4N 3M5, Canada; 4 Child Health Evaluative Sciences, The Hospital for Sick Children, Toronto, ON M5G 1X8, Canada; 5 Faculty of Science, University of Waterloo, Waterloo, ON N2L 3G1, Canada; 6 Faculty of Health, University of Waterloo, Waterloo, ON N2L 3G1, Canada; 7 Faculty of Engineering, University of Waterloo, Waterloo, ON N2L 3G1, Canada

**Keywords:** health-related quality of life (HRQoL), EQ-5D-3L, implementation, patient experience

## Abstract

Cancer treatments impact health-related quality of life (HRQoL). EQ-5D is a standardized generic measure of HRQoL. The objective of this project was to assess, from the patient’s perspective, the feasibility of implementing real-world EQ-5D-3L measurement at a pilot site, as a first step to large-scale collection of EQ-5D from patients with cancer across Ontario. This was a prospective longitudinal study at a single oncology centre to understand the feasibility of routinely collecting EQ-5D-3L while patients receive chemotherapy (N = 170). Consenting patients completed an additional questionnaire on feasibility, and a subset of participants were directly interviewed to provide further feedback and suggest improvements to questionnaire collection. Themes emerging from the interviews were analyzed using content analysis. Of 170 eligible and consenting patients who completed an initial EQ-5D-3L questionnaire, 103 (60.6%) completed at least one follow-up questionnaire. When asked about willingness to answer future questionnaires at subsequent visits, 115 (67.3%) answered definitely; 35 (20.5%) very likely. Patients provided feedback on their overall experience of completing EQ-5D-3L, the questionnaire presentation, frequency of completion, and analytic plans. Patients expressed that routinely collecting EQ-5D-3L is feasible. Incorporating patient feedback regarding EQ-5D collection will facilitate implementation of systematic collection at cancer centres across Ontario.

## 1. Introduction

Cancer treatments impact health-related quality of life (HRQoL) [1,2]. HRQoL is typically measured using questionnaires such as the EQ-5D-3L that ask respondents about dimensions of HRQoL [3]. Knowing patient preferences for important aspects of HRQoL is essential when two or more treatments have similar survival outcomes, but may differ in adverse effects [4]. HRQoL measurements with preference-based scoring algorithms can be converted to health utilities (HUs) which are a quantitative measure, anchored at 0 (dead) and 1 (full health). The product of HUs and length of life produces quality adjusted life years (QALYs), which are a key input in economic evaluations, including cost utility analyses, which are routinely used to inform drug funding decisions. HUs can measure patient preferences at three broad levels in cancer care: (1) micro (e.g., clinical decision-making), (2) meso (e.g., economic analyses), (3) macro (e.g., population health) levels [5]. The most common application of HUs is in economic models [6], and the validity of the economic model depends directly on the utility measurement.

EQ-5D-3L health utilities used in economic models are commonly derived from clinical trials, yet there are gaps in inclusion and reporting of HRQoL endpoints in phase III trials. A literature review found that only 35% of oncology clinical trials between 2017–2021 analyzed and reported HRQoL outcomes [7]. The proportion of phase III oncology trials including HRQoL outcomes increased for more recent publications (68% of 388 publications in 2017–2021 vs. 53% of 446 publications in 2012–2016) [7]. Of the HRQoL outcomes reported in clinical trials, an important limitation is that they may inadequately represent real-world HUs. Clinical trials have strict inclusion and exclusion criteria, therefore patients in clinical trials are highly selected, high-functioning, and receive closer monitoring, atypical of the real-world cancer population [8,9]. In Ontario, there is no routine collection of HRQoL from patients with cancer, even though existing infrastructure at Ontario Health-Cancer Care Ontario (OH-CCO) could facilitate collecting patient-reported outcomes routinely [10].

In 2015, OH-CCO recommended the systematic collection and monitoring of patients’ HRQoL during systemic therapies, to evaluate their value [10]. Routine system-wide collection of HRQoL HUs could facilitate clinical decision-making between patients and clinicians, and facilitate real-world economic evaluations of publicly funded cancer therapies to support health system sustainability. The objective of this project was to understand, from the patient’s perspective, the feasibility of prospectively collecting real-world EQ-5D-3L at a pilot site as a first step to large-scale collection of EQ-5D HUs across Ontario.

## 2. Materials and Methods

### 2.1. Study Population, Design, and Setting

Eligible patients were 18 years and over, starting any publicly reimbursed systemic therapy, with histologically confirmed malignancy, who provided informed consent. A prospective convenience sample of patients with any solid tumour or hematological malignancy was surveyed to complete the EQ-5D-3L during chemotherapy appointment at the Sunnybrook Odette Cancer Centre. Patients were accrued from May to November 2024 and followed up until February 2025. The three-level version of EQ-5D was collected because this version has more historic use than the 5L version as a clinical outcome assessment in health technology assessment, regulatory reviews, and systematic literature reviews [11]. Measuring EQ-5D-3L allows for direct future comparisons of clinical trial and real-world EQ-5D-3L health utilities when conducting real-world cost–utility analyses.

### 2.2. Patient Accrual

Four research coordinators (RD, SC, EZ, CY) recruited and/or followed up with patients from the chemotherapy unit throughout the week, at different times of day, to capture a range of patient responses. Patients were screened for eligibility prior to their appointments using an appointment scheduling system. If a patient was eligible for our study, they were approached during their initial chemotherapy appointment. If a patient consented to participate in the study, they were presented with the following forms: the EQ-5D-3L, the EQ-VAS for REDCap and paper administration (Supplementary File S1), and brief optional questions on the feasibility of completing the EQ-5D-3L, participant demographics, and diagnosis (Supplementary File S2).

### 2.3. Questionnaire Version and Data Capture

EQ-5D-3L was the primary outcome measure used in this study. The instrument comprises five dimensions: mobility, self-care, usual activities, pain/discomfort, anxiety/depression [12,13], each with three levels: no problems, some problems, or extreme problems/unable to perform activities [3]. The EQ-5D visual analogue scale (VAS) was also administered, which is a self-assessed measure of overall health on a scale from 0 (the worst health imaginable) to 100 (the best health imaginable) [14]. The EQ-5D instruments are well-established health utility instruments for measuring HRQoL in oncology with test-retest reliability, convergent validity, and known-group validity in six common cancers [15].

We provided EQ-5D-3L proxy version 1 in English for individuals unable to complete their own EQ-5D-3L responses. Proxy version 1 is completed by a caregiver proxy who rates the patient’s HRQoL according to the proxy’s opinion [16]. All patient responses were entered into REDCap^R^, a secure web-based application for managing online surveys and databases commonly used in clinical trials.

### 2.4. Analysis of Feasibility

#### 2.4.1. Questionnaires

The primary focus of this study was the feasibility of collecting EQ-5D-3L, a component of micro-level evaluation. Descriptive statistics assessing feasibility were the following:

- percentage of consenting and eligible patients who completed at least one EQ-5D-3L questionnaire [17];
- rating of patient’s willingness to answer future EQ-5D-3L [17,18].

#### 2.4.2. Interviews

Consenting patients were invited to participate in an interview with two interviewers (two-on-one) to provide feedback on the pilot implementation process to inform overall feasibility. Patients were interviewed to understand their perspectives on the following points:

1. Their overall experience with completing the EQ-5D-3L;
2. How the questionnaire is presented (e.g., tablet, paper), and the layout of the questions;
3. How often they are asked to complete the EQ-5D-3L;
4. How the EQ-5D-3L data will be analyzed.

Two researchers conducted the interviews (EZ and RM). Patient interviews were recorded using an online platform (Zoom) and transcribed verbatim. Interview transcripts were analyzed using content analysis by EZ, RM, and TT. A code book of themes was created as themes emerged. A copy of the interview guide used is available as Supplementary File S2.

### 2.5. Licenses

An academic license to administer the EQ-5D-3L was obtained from the EuroQol Foundation.

## 3. Results

### 3.1. Patients

Table 1 summarizes baseline characteristics of study participants. Of those who completed this voluntary questionnaire (responses per question: N = 157 to 168), patients were 56.5% female, with 65.3% of patients between ages 50 and 74 years. The majority of patients attended college or university (74.7%). The top five primary cancers were (%): gynecological (23.5%), head and neck (18.8%), breast (10.6%), hematological (10.0%), and upper gastrointestinal (7.6%).

The most common reasons eligible patients did not complete EQ-5D-3L questionnaires were (1) cancelled appointments, (2) patient was missed by the coordinator, (3) patient was busy when approached, or (4) patient was sleeping when approached. Other challenges faced by study coordinators included difficulty connecting with patients who have short treatment duration (<60 min), and language barriers with patients with limited fluency in English.

Out of 57 patients who expressed a willingness to be contacted for an individual interview to provide additional feedback on the EQ-5D-3L, nine interviews were completed (7 female, 2 male).

### 3.2. Questionnaire

**Figure 1:**
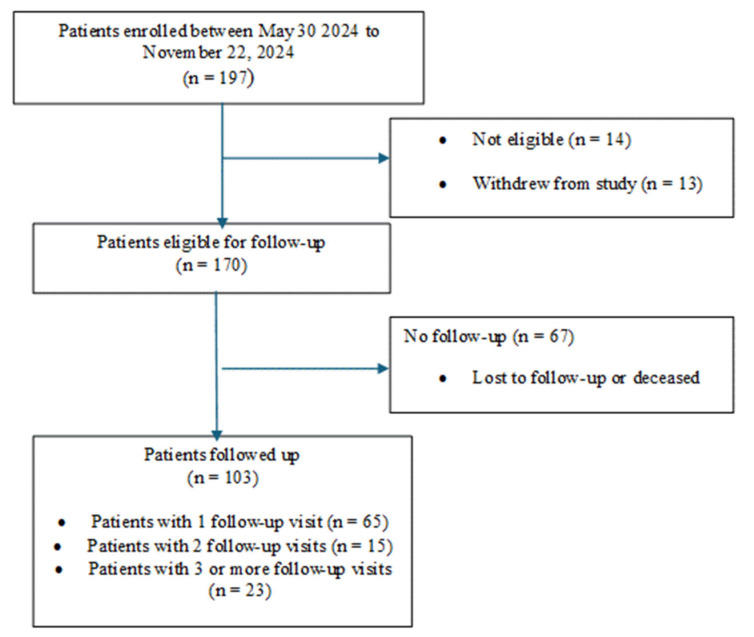
Flow diagram of patient enrollment and follow-up.

Figure 1 is a diagram of patient enrollment and follow-up. Within an accrual period of 8 months, 103 (60.6%) out of 170 eligible and consenting patients were followed up at least once. Sixty-five (63.1%) patients had one follow-up visit, 15 (14.6%) had two follow-up visits, and 23 (22.3%) had three or more follow-up visits. Of the 170 enrolled patients, 160 (94.1%) completed an optional feasibility and demographic questionnaire during their initial visit.

**Figure 2:**
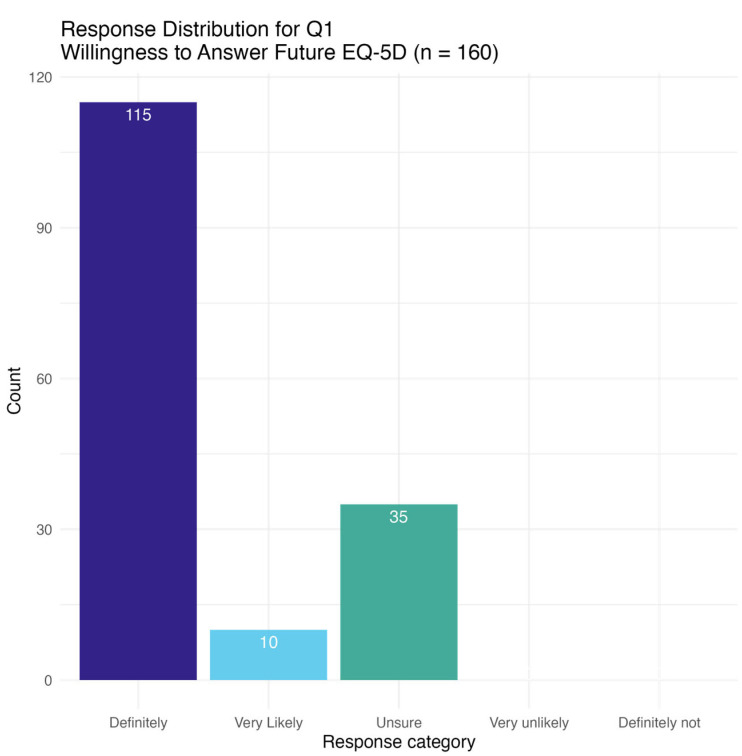
Patient willingness to continue to answer EQ-5D questions at future clinic visits. Q1. “Are you willing to continue to answer EQ-5D questions at each clinic visit?”; There were no patient responses in “very unlikely”, and “definitely not”.

Figure 2 is a histogram of response distributions to Question 1, “Are you willing to continue to answer EQ-5D questions at each clinic visit?” Of patients who agreed to routinely complete the EQ-5D-3L at subsequent visits, the number of patients expressing a willingness to complete questionnaires was 115 (71.9%) definitely, and 35 (21.9%) very likely.

**Figure 3:**
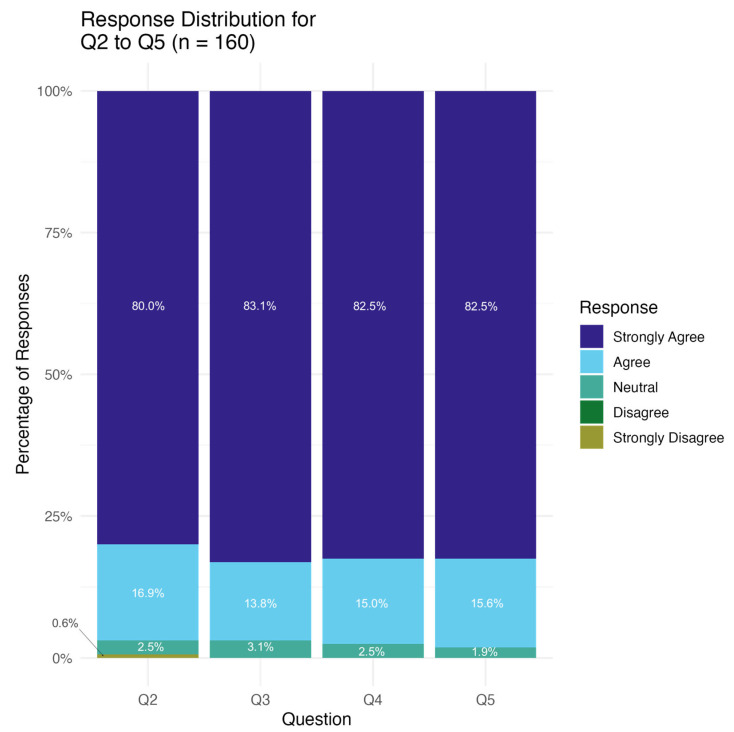
Patient responses regarding ease of comprehension, ease of completion, acceptability of the questions, and length of the questions: Q2. All instructions and questions were easy to understand; Q3. It was easy for me to complete the questionnaires; Q4. The questions asked were acceptable to me; Q5. The length of the questions was acceptable to me. There were no responses for “disagree” in all questions. Only 0.6% of respondents to Q2 selected “strongly disagree”, and there were no “strongly disagree” responses to Q3 to Q5.

Figure 3 shows stacked bar graphs of Questions 2 to 5 that cover topics about the EQ-5D-3L on ease of comprehension, ease of completion, acceptability of the questions, and length of the questions, respectively. When asked for input about the EQ-5D questionnaire, most patients (128, (80.0%)) selected “strongly agree” that the EQ-5D-3L instructions and questions are easy to understand, with 27 (16.9%) selecting agree, four (2.5%) selecting “neutral”, and one (0.6%) selecting “strongly disagree” (Question 2: “All instructions and questions were easy to understand”). No patients identified difficulty completing the questionnaire, with 83.1% (133) selecting that they “strongly agree” the questionnaire was easy for them to complete, 22 (13.8%) selecting agree, and five (3.1%) selecting neutral (Question 3: ”It was easy for me to complete the questionnaires”). Further, the questions themselves were seen as acceptable with participants indicating that they “strongly agree” (132, 82.5%), “agree” (24, 15.0%), or were “neutral” (four, 2.5%) that questions asked were acceptable to them (Question 4: “The questions asked were acceptable to me”). Lastly, participants were satisfied with the length of the questionnaire, with 132 (82.5%) “strongly agree”, 25 (15.6%) “agree”, and three (1.9%) “neutral” that the length was acceptable to them (Question 5: “The length of the questions was acceptable to me”).

### 3.3. Interviews

Table 2 presents the key themes from our interviews along with illustrative quotes. An elaboration of each of the themes is outlined below.

### 3.4. Overall Experience

Patients overall reported the questionnaire was simple to understand and quick to complete (See Table 2). Patients also commented on the EQ-5D-3L questions being limited, and not representative of the spectrum of their experience with cancer and its treatments.

Administering the EQ-5D-3L in the chemotherapy unit had both positive and negative aspects. For some patients, this was seen as an opportunity as they had time and were open to being approached to complete a questionnaire. For other patients, it was challenging because they were receiving chemotherapy or other interventions simultaneously (e.g., antihistamines). Patients also pointed out their responses were influenced by moment-to-moment changes to therapy.

*“If I were in the waiting room… Why are you asking me these questions now if in 2 h I have an allergic reaction, which I did one time”.*

*“it’s fair to do it. I do think that in the waiting room would be worse than at the end of Chemo”.*

Patients provided feedback on suggested timing for administering the EQ-5D-3L, other than during chemotherapy. Ideal times ranged from before chemotherapy, two weeks after chemotherapy, or at the end of chemotherapy.

Patients had different preferences for the timing within their chemotherapy visit to complete the EQ-5D-3L. One suggestion was to send the EQ-5D-3L to patients to complete the questionnaire in their own time. Others preferred having an in-person administration as they could ask clarification questions:

*“The one benefit of the approach was that you were there in person, so if I had a request for clarifying question, I could ask in the moment versus when you do something online, then it is it’s truly open to interpretation”.*

The overall sentiment was this questionnaire was simple, short, and feasible to administer within the chemotherapy unit.

#### 3.4.1. How the Questionnaire Is Presented

Patients were generally satisfied with a paper copy of the EQ-5D-3L, while others suggested that an electronic option could be useful. Those who preferred a paper copy of the EQ-5D-3L cited reasons including it being easier to read by themselves, and easier comprehension. Individuals who preferred to receive EQ-5D-3L by electronic format described greater flexibility in the timing of completing the EQ-5D-3L around their chemotherapy appointment, while in the waiting room, or having an option to complete it on their own phone.

All patients expressed satisfaction with the layout or formatting of the questionnaire. Patients appeared to like the three response options of the EQ-5D-3L. A participant mentioned that a five-level version could be more useful.

#### 3.4.2. Frequency of Administering Questions

Patients made a range of suggestions on the most suitable frequency of administering the EQ-5D-3L. This ranged from every visit, every 2–3 weeks, to visits throughout their systemic therapy journey (beginning, middle, end). Patients did point out that the day of receiving chemotherapy is an inadequate snapshot of their HRQoL (Table 2).

#### 3.4.3. How to Analyze and Interpret Data

Patients varied in their input on analysis and interpretation of EQ-5D-3L data. Patients suggested considering participant age, institution, and mental health concerns when analyzing data. External influences were acknowledged on their HRQoL (e.g., the weather, traffic). Other patients found it challenging to suggest topics of analysis, but several patients suggested that EQ-5D-3L responses could inform them about how others on a similar treatment regimen to theirs have felt.

## 4. Discussion

We found that routinely collecting EQ-5D-3L from the Sunnybrook Odette Cancer centre is feasible with 103 (60.6%) of consenting and eligible patients completing at least one follow-up questionnaire. When asked whether patients are willing to complete the EQ-5D-3L questionnaire at subsequent visits, 115 (71.9%) answered definitely and 35 (21.9%) answered very likely. Patient feedback on their experience of completing the EQ-5D-3L while receiving systemic therapy was favourable overall. Patients also provided suggestions on suitable timing to complete the EQ-5D, centred around their systemic therapy (before onset, 2 weeks after, or at the end of treatment). Patients were split in their preference on receiving the EQ-5D in paper format with the opportunity to ask questions, versus a self-directed electronic format.

From a micro perspective, the percentage of patients who agreed to complete subsequent EQ-5D-3L questionnaires was comparable to a previous EQ-5D-5L study in breast cancer only [17]. The feasibility of collecting EQ-5D-5L or EQ-5D-3L has been demonstrated in patients with lymphoma (EQ-5D-5L) [19], and lung cancer (EQ-5D-3L) [20]. A systematic literature review found that EQ-5D-3L had good feasibility properties amongst individuals over 65 years, even though individuals who are elderly have lower feasibility than individuals who are younger [21]. Individuals who are elderly are more likely to require assistance when completing the EQ-5D and are more likely to prefer an interviewer-based approach [21]. Feasibility of administering the EQ-5D-3L has been investigated in a mixed methods study as part of patients’ self-management support program for patients with esophageal cancer [22], or as part of an advance care planning intervention for patients with stage III/IV cancer and their caregivers. In these examples, the respective programs were feasible and had high levels of patient compliance, yet were accompanied by their own unique challenges [21,22] that were different from our study. These programs had different objectives, included multiple questionnaires, and comprised different samples of patients with cancer, making these results not directly comparable with our study.

We adopted a phase-based approach to implementing EQ-5D that drew on lessons learned from OH-CCO’s initial collection of patient-reported outcomes using the *Your Symptoms Matter platform* [23]. This approach is well-aligned with Ernst et al.’s early adoption stage framework which implements patient-reported outcome measures across health care systems by starting with pilot sites [24]. The feasibility of our pilot implementation provides a template for future large-scale implementation using the existing “Your Symptoms Matter” platform at all cancer centres across Ontario.

There are examples of real-world collection of EQ-5D at specific oncology centres, at the provincial level in Alberta, and nationally by the Canadian Institute for Health Information (CIHI). Moskovitz et al. cited an unpublished pilot survey of a convenience sample of patients with cancer (n = 100), finding that 83% of patients were willing to routinely complete patient-reported outcomes, yet only 42% were willing to answer repetitive or duplicative questions, inevitably the case when asked to answer multiple overlapping questionnaires [18].

Alberta Health Services has endorsed EQ-5D-5L as the provincial HRQoL tool, and Cancer Care Alberta collects data on an ad hoc basis for performance planning and economic analyses, with planning underway for routine collection [25]. Micro (person-centered care), meso (clinic), and macro (program) levels of reporting have been achieved with digital dashboards. To achieve this, patient-reported outcomes are incorporated into routine patient electronic medical records, resulting in improved patient-clinician communication, earlier problem detection, symptom management, and improvement of patient outcomes [25]. This Cancer Care Alberta initiative demonstrated that province-wide collection of patient-reported outcomes is possible, and produces benefits across multiple levels of care.

At the national level, CIHI currently collects EQ-5D-5L amongst its hip and knee replacement patients [26], yet there is no collection of EQ-5D amongst patients with cancer. When implementing EQ-5D collection at Ontario cancer centres, patients can be informed in advance that overlapping questions between EQ-5D and other questions already routinely collected on the *Your Symptoms Matter* platform are intended to assess for patient understanding and response consistency [27]. Learning from Alberta, and other jurisdictions, our study is a key initial step to scaling up EQ-5D collection amongst all oncology centres within Ontario.

This study has several strengths. We recruited a diverse sample of patients with a range of cancer diagnoses, therefore our EQ-5D-3L findings are representative of a general population of patients with cancer. We utilized multiple methods including incorporating patient-rated willingness to complete subsequent EQ-5D-3L questionnaires and conducting semi-structured interviews with patients to understand the patient experience. Patient perceptions of the strengths of the EQ-5D-3L were that it is simple, easy to understand, and quick to complete. Most patients had no issues with the layout or formatting of the questionnaire.

The main weakness of this study is that it was conducted at a single oncology site, which limits the generalizability to other sites. This study has demonstrated initial promise, particularly feasibility at this single site. This feasibility would need to be confirmed more broadly at the provincial level before province-wise implementation of the EQ-5D instrument. Acknowledging that the pilot site may differ from other sites, this presents an opportunity to identify customizable solutions across sites in order to improve the patient experience. The main challenge of the EQ-5D-3L questionnaire is how the questions are limited in capturing the range of HRQoL experiences in patients undergoing cancer treatments. Implementing the EQ-5D questionnaire on OH-CCO’s *Your Symptoms Matter* platform would mitigate this issue in part by combining other patient-reported outcome measures with EQ-5D responses, including the Edmonton Symptom Assessment System (ESAS) already in place. The response options and the setting of completing the questionnaire in the chemotherapy unit were seen by patients as both a strength and a weakness. The three levels of the EQ-5D-3L limited the response options, which is potentially positive; one respondent mentioned that the five-level option would be more useful. Our selection of EQ-5D-3L instead of EQ-5D-5L considered existing studies that have collected EQ-5D-3L [11], and potential comparisons with existing clinical trials. Mapping algorithms also allow conversion between EQ-5D-3L and EQ-5D-5L HUs [28,29]. Because the EQ-5D-5L instrument overall shows improved performance and sensitivity than the EQ-5D-3L instrument [30,31], we believe it will be more appropriate to use the 5L version for broader implementation, to obtain more precise measurement of HRQoL. Another weakness of this in-person study was the relatively high coordinator burden, which led to cumbersome recruitment and difficulty identifying appointment times for follow-ups. Future researchers are advised to include multiple administration forms, including electronic and paper options. We anticipate that this will alleviate some coordinator capacity, while allowing patients to choose their preferred method of answering the questionnaire, thus encouraging more follow-up completion. It will be important to further determine the acceptability of an electronic version of the questionnaire among patients to better inform the scale-up design of province-wide collection in terms of balancing accessibility, cost, and coordinator burden.

Completing the questionnaire in the chemotherapy unit was seen as both an opportunity and a challenge—while patients had the time to respond while they were receiving ongoing treatments, some found the timing sub-optimal since they were focused on the interventions they were receiving. To mitigate the potential impact of moment-to-moment changes in the patient’s condition, future implementation strategies could include encouraging patients to complete the questionnaire during a window of time before they commence their clinic visit, and/or on specific days of their chemotherapy regimen.

We have several future directions. A future study will conduct additional analyses on data collected from this study to understand how inequities predict EQ-5D-3L health utilities. We also have plans to consult with interested parties, namely leadership at the Sunnybrook Odette Cancer Centre, and interested external organizations including OH-CCO and CIHI. Our future steps include scaling up the collection of EQ-5D across all Ontario Health regions.

## 5. Conclusions

This study demonstrated the feasibility of collecting EQ-5D-3L amongst patients with a range of cancers receiving chemotherapy at a pilot oncology centre. The feasibility of collecting EQ-5D-3L across all cancers is likely generalizable to the collection of EQ-5D-5L in the future, given other positive feasibility studies of collecting EQ-5D-5L alone, or as part of other questionnaires. Themes emerging from our interviews provided actionable steps to consider when scaling up EQ-5D-5L collection across Ontario.

## Figures and Tables

**Table 1:** Baseline characteristics of study participants.

	Study Participants (N = 170)
	Number (%)
Sex	
Male	71 (41.8)
Female	96 (56.5)
Not disclosed	3 (1.8)
Age	
<50	23 (13.5)
50 to 74	111 (65.3)
75 to 99	32 (18.8)
Not disclosed	4 (2.4)
Education	
Did not attend College or University	38 (22.4%)
Attended College or University	127 (74.7)
Prefer not to answer	3 (1.8)
Not disclosed	2 (1.2)
Primary Cancer Site	
Breast	18 (10.6%)
Central nervous system	1 (0.6%)
Colorectal	12 (7.1%)
Genitourinary	5 (2.9%)
Gynecological	40 (23.5%)
Head and Neck	32 (18.8%)
Hematological	17 (10.0%)
Melanoma	7 (4.1%)
Skin	2 (1.2%)
Thoracic	12 (7.1%)
Upper gastrointestinal	13 (7.6%)
Not disclosed	11 (6.5%)
Another current or past primary cancer in last 5 years	
Yes	19 (11.2%)
No	148 (87.1%)
Not disclosed	3 (1.8%)

**Table 2:** Themes emerging from patient interviews.

Theme	Sub-Theme and Selected Quotes
Overall experience	
**Straightforward questionnaire**	“it’s probably the simplest questionnaire I’ve had to fill out”.
**Quick completion**	“it was simple and fast”. “It only takes a few minutes to fill out the questionnaire… But it still gives you guys an idea of how everybody’s doing, and I think it’s great”.
**Clinic setting**	Positives “I had time on my hands. It was fine”. Negatives Changeability, timing “Why are you asking me these questions now? How does this reflect today, if in 2 h, I have an allergic reaction which I did one time”.
**Questionnaire content**	
***Oversimplified HRQoL***	“I think it was limited to tell you the truth… and it was too fast to capture the pain that we go through, or the discomfort we go through”. “I guess it’s just when I when I know someone’s gonna talk to me about my quality of life and it boils down to this, I’m like, oh, there’s just so much more. That is my quality of life that I don’t even. What’s this really going to tell you?”
***Suggestions for questions to include***	Should have optional free text box after every question “what I found with my previous chemo now with this one that I was also doing radiation because I couldn’t eat and I couldn’t drink. So that became a real source of stress and anxiety for me as a patient”.
Administration form	
**Electronic-questionnaire sent to patient**	“So if you had the ability something electronically. So you know, a quick text that you could do it like in 2 min on your phone”. “I like the idea of it being sent to you versus you having to go on like go online or do it at the hospital when you’re all you know running around”.
**Paper**	“I’d rather see the paper copy and read it myself. So I could understand it better”. “I’m 63. So if you ask me if I prefer paper or electronic chances are I’m going to say, paper”.
Layout	
***Facilitators***	
**No concerns**	“…and the layout is equally simple. And there’s a lot of value in that kind of simplicity, because it makes it accessible for a much broader audience”. “I think it was done very well. It was easy for me to read and easy for me to fill out, so I don’t have any feedback regarding changing it. I think it was great”.
***Barriers***	
**Small font**	“The font is small. Well, on my screen. It’s small”. “maybe a little bigger would be good”.
**Response options**	Satisfaction with 3 levels “it’s good to have just 3 answers to the questions, too. So you don’t have a lot to to look at”. Five-point scale could be useful
Frequency of administration	Beginning, middle, end “Maybe do one in the beginning, one in the middle, one in the end of their treatment. So then you have the rough estimate”.
Data analysis	Influence of the environment Mental health Hospital/institute-specific HRQoL Age
Interpretation of results	
	Knowing what to expect “I would wanna know that it was gonna be bad. and then then but it’s gonna be better”. “quality of life like it’s just good to know if other people are taking the same medications that they’re like functioning as well as I can be, so that I can still do all the things that I have to do”. “It’s it’s very reassuring to know that, you know, if I if I go to a doctor with certain symptoms and say, Oh, yeah, that’s…that’s very common”.
	Adaptation “the scale is quite vast. So for somebody like me, I automatically go to the top end of the scale. Even though I have cancer. And somebody says, Well, I’m feeling at 50 because I have cancer. But yet I can do everything. But I feel good in my life. You know what I mean, where I am”.

## Data Availability

The raw data supporting the conclusions of this article can be made available by the authors on request.

## Supplementary Materials

The following supporting information can be downloaded at: https://www.mdpi.com/article/10.3390/curroncol32060308/s1, Supplementary File S1: Participant demographic questionnaire, Supplementary File S2: Patient Interview Guide.

## Abbreviations

The following abbreviations are used in this manuscript:

CIHI	Canadian Institute for Health Information
ESAS	Edmonton Symptom Assessment Scale
HRQoL	Health-related quality of life
HUs	Health utilities
ISAAC	Interactive Symptom Assessment and Collection
OH-CCO	Ontario Health-Cancer Care Ontario
QALY	Quality-adjusted life year
VAS	Visual analogue scale
YSM	Your Symptoms Matter

## References

[1] Boini S., Briancon S., Guillemin F., Galan P., Hercberg S. (2004). Impact of cancer occurrence on health-related quality of life: A longitudinal pre-post assessment. Health Qual. Life Outcomes.

[2] Han X., Robinson L., Jensen R., Smith T., Yabroff K. (2021). Factors Associated With Health-Related Quality of Life Among Cancer Survivors in the United States. JNCI Cancer Spectr..

[3] EuroQol Foundation (2018). EQ-5D-3L User Guide.

[4] Wennberg J.E., Fisher E.S., Skinner J.S. (2002). Geography and the debate over Medicare reform. Health Aff..

[5] Lipscomb J., Donaldson M.S., Hiatt R.A. (2004). Cancer outcomes research and the arenas of application. J. Natl. Cancer Inst. Monogr..

[6] Canadian Agency for Drugs and Technologies in Health (2017). CADTH Methods and Guidelines: Guidelines for the Economic Evaluation of Health Technologies: Canada.

[7] Marandino L., Trastu F., Ghisoni E., Lombardi P., Mariniello A., Reale M.L., Aimar G., Audisio M., Bungaro M., Caglio A. (2023). Time trends in health-related quality of life assessment and reporting within publications of oncology randomised phase III trials: A meta-research study. BMJ Oncol..

[8] Booth C.M., Tannock I.F. (2014). Randomised controlled trials and population-based observational research: Partners in the evolution of medical evidence. Br. J. Cancer.

[9] Kalata P., Martus P., Zettl H., Rodel C., Hohenberger W., Raab R., Becker H., Liersch T., Wittekind C., Sauer R. (2009). Differences between clinical trial participants and patients in a population-based registry: The German Rectal Cancer Study vs. the Rostock Cancer Registry. Dis. Colon. Rectum.

[10] Ontario Cancer Plan IV. https://www.cancercareontario.ca/sites/ccocancercare/files/assets/CCOOntarioCancerPlan4.pdf.

[11] Shaw C., Longworth L., Bennett B., McEntee-Richardson L., Shaw J.W. (2024). A Review of the Use of EQ-5D for Clinical Outcome Assessment in Health Technology Assessment, Regulatory Claims, and Published Literature. Patient.

[12] Dolan P., Gudex C., Kind P., Williams A. (1995). A Social Tariff for EuroQol: Results from a UK General Population Survey.

[13] Herdman M., Gudex C., Lloyd A., Janssen M., Kind P., Parkin D., Bonsel G., Badia X. (2011). Development and preliminary testing of the new five-level version of EQ-5D (EQ-5D-5L). Qual. Life Res..

[14] Devlin N., Parkin D., Janssen B. (2020). Methods for Analysing and Reporting EQ-5D Data.

[15] Zeng X., Sui M., Liu B., Yang H., Liu R., Tan R.L., Xu J., Zheng E., Yang J., Liu C. (2021). Measurement Properties of the EQ-5D-5L and EQ-5D-3L in Six Commonly Diagnosed Cancers. Patient.

[16] EuroQol Foundation EQ-5D Available Versions. https://euroqol.org/register/obtain-eq-5d/available-versions/#:%7E:text=EQ%2D5D%2D5L%20Interviewer%20Administered%20Proxy%20version%202%3A%20Developed,complete%20a%20paper%2Fdigital%20questionnaire.

[17] Torres S., Bayoumi A.M., Abrahao A.B.K., Trudeau M., Pritchard K.I., Li C.N., Mitsakakis N., Liu G., Krahn M. (2024). Implementing routine collection of EQ-5D-5L in a breast cancer outpatient clinic. PLoS ONE.

[18] Moskovitz M., Jao K., Su J., Brown M.C., Naik H., Eng L., Wang T., Kuo J., Leung Y., Xu W. (2019). Combined cancer patient-reported symptom and health utility tool for routine clinical implementation: A real-world comparison of the ESAS and EQ-5D in multiple cancer sites. Curr. Oncol..

[19] Xu R.H., Wong E.L., Jin J., Huang H., Dong D. (2021). Health-related quality of life measured using EQ-5D in patients with lymphomas. Support. Care Cancer.

[20] Yorke J., Lloyd-Williams M., Smith J., Blackhall F., Harle A., Warden J., Ellis J., Pilling M., Haines J., Luker K. (2015). Management of the respiratory distress symptom cluster in lung cancer: A randomised controlled feasibility trial. Support. Care Cancer.

[21] Marten O., Brand L., Greiner W. (2022). Feasibility of the EQ-5D in the elderly population: A systematic review of the literature. Qual. Life Res..

[22] King E., Algeo N., Connolly D. (2023). Feasibility of OptiMaL, a Self-Management Programme for Oesophageal Cancer Survivors. Cancer Control.

[23] Basch E., Barbera L., Kerrigan C.L., Velikova G. (2018). Implementation of Patient-Reported Outcomes in Routine Medical Care. Am. Soc. Clin. Oncol. Educ. Book..

[24] Ernst S.K., Steinbeck V., Busse R., Pross C. (2022). Toward System-Wide Implementation of Patient-Reported Outcome Measures: A Framework for Countries, States, and Regions. Value Health.

[25] Watson L., Delure A., Qi S., Link C., Chmielewski L., Photitai E., Smith L. (2021). Utilizing Patient Reported Outcome Measures (PROMs) in ambulatory oncology in Alberta: Digital reporting at the micro, meso and macro level. J. Patient Rep. Outcomes.

[26] Canadian Institute for Health Information CIHI’s PROMs Program. https://www.cihi.ca/en/patient-reported-outcome-measures-proms/cihis-proms-program.

[27] BMJ (2025). Chapter 5. Planning and conducting a survey. Epidemiology.

[28] Pickard A.S., Kohlmann T., Janssen M.F., Bonsel G., Rosenbloom S., Cella D. (2007). Evaluating equivalency between response systems: Application of the Rasch model to a 3-level and 5-level EQ-5D. Med. Care.

[29] van Hout B.A., Shaw J.W. (2021). Mapping EQ-5D-3L to EQ-5D-5L. Value Health.

[30] Janssen M.F., Bonsel G.J., Luo N. (2018). Is EQ-5D-5L Better Than EQ-5D-3L? A Head-to-Head Comparison of Descriptive Systems and Value Sets from Seven Countries. Pharmacoeconomics.

[31] Janssen M.F., Buchholz I., Golicki D., Bonsel G.J. (2022). Is EQ-5D-5L Better Than EQ-5D-3L Over Time? A Head-to-Head Comparison of Responsiveness of Descriptive Systems and Value Sets from Nine Countries. Pharmacoeconomics.

