# Supplementary material for: Patient Experiences Regarding Feasibility of Implementing Real-World EQ-5D Collection at an Oncology Centre in Ontario, Canada
Source: Curr Oncol. 2025 May 27;32(6):308. doi: 10.3390/curroncol32060308 (PMC12191542; doi:10.3390/curroncol32060308)
Supplement: Supplementary file 1 [file curroncol-32-00308-s001.zip › Supplementary File S1 Demographic form.pdf]

Participant ID:

Participant Feedback Form

Please complete the following questions about:
A. Completing the EQ-5D questionnaire,
B. Your demographic information, and
C. Your diagnosis.

A. Completing the EQ-5D questionnaire

We value your feedback regarding the questionnaire you just answered.

1. Are you willing to continue to answer EQ-5D questions at each clinic visit?

- ☐ Definitely
- ☐ Very likely
- ☐ Unsure
- ☐ Very unlikely
- ☐ Definitely not

If you have agreed to continue participating, please provide your email and/or phone number so that we can contact you later for follow-up EQ-5D questionnaires:

Email: ____

Telephone: ____

2. For the next series of questions, please select the response that best describes your opinion:

a) All instructions and questions were easy to understand.

- ☐ Strongly agree
- ☐ Agree
- ☐ Neutral
- ☐ Disagree
- ☐ Strongly disagree

b) It was easy for me to complete the questionnaires.

- ☐ Strongly agree
- ☐ Agree
- ☐ Neutral
- ☐ Disagree
- ☐ Strongly disagree

c) The questions asked were acceptable to me.

☐ Strongly agree
☐ Agree
☐ Neutral
☐ Disagree
☐ Strongly disagree

d) The length of the questions was acceptable to me.

☐ Strongly agree
☐ Agree
☐ Neutral
☐ Disagree
☐ Strongly disagree

e) If you have any additional comments or suggestions about the EQ-5D questionnaire, please write your response in the textbox below.

f) Are you interested in being contacted for a one-on-one interview to discuss your experience with completing this questionnaire?

☐ Yes
☐ No

If you have selected **Yes**, then please include your preferred method of contact to schedule an interview time:

Email: ____

Telephone: ____________________________________

B. Demographic questions

1. What is your date of birth?

(YYYY/MM/DD): ______________________________

2. What is your sex at birth?

☐ Female
☐ Male
☐ Other

- ☐ Prefer not to answer

3. What is your gender?

- ☐ Woman
- ☐ Man
- ☐ Other
- ☐ Prefer not to answer

4. Were you born in Canada?

- ☐ Yes
- ☐ No
- ☐ I don't know
- ☐ Prefer not to answer

5. Which of the following categories best describes your race or racial background? Please select all that apply:

- ☐ Black (e.g., African, African Canadian, Afro-Caribbean descent)
- ☐ East Asian (e.g., Chinese, Japanese, Korean, Taiwanese descent)
- ☐ Indigenous (e.g., First Nations, Inuk/Inuit, Métis)
- ☐ Latin American (e.g., Hispanic or Latin American descent)
- ☐ Middle Eastern (e.g., Arab, Persian, West Asian descent (Afghan, Egyptian, Iranian, Kurdish, Lebanese, Turkish))
- ☐ South Asian (e.g., Bangladeshi, Indian, Indo-Caribbean, Pakistani, Sri Lankan)
- ☐ Southeast Asian (e.g., Cambodian, Filipino, Indonesian, Thai, Vietnamese, or other Southeast Asian descent)
- ☐ White (e.g., European descent)
- ☐ Other (please specify): ____________________
- ☐ Do not know
- ☐ Prefer not to answer

6. What is your education level? Please select one option:

- ☐ Grade 8 or under
- ☐ Attended or graduated high school
- ☐ Attended or graduated college or university
- ☐ Postgraduate or professional
- ☐ Prefer not to answer

7. What is your marital status? Please select one option:

- ☐ Married or common law
- ☐ Separated or divorced
- ☐ Widowed

- ☐ Single or never married
- ☐ Prefer not to answer

8. What is your employment status? Please select one option.

- ☐ Retired
- ☐ Working full-time
- ☐ Working part-time
- ☐ Unemployed
- ☐ Other (please specify): ________________
- ☐ Prefer not to answer

9. What was your total family income before taxes this year? Please select one option.

- ☐ $0 to $29,999
- ☐ $30,000 to $59,999
- ☐ $60,000 to $89,999
- ☐ $90,000 to $119,999
- ☐ $120,000 to $149,999
- ☐ $150,000 or more
- ☐ Do not know
- ☐ Prefer not to answer

C. Your diagnosis

10. Current primary cancer diagnosis for which you are receiving treatment:

____

11. Another current or past primary cancer in the last 5 years:

- ☐ Yes
- ☐ No

If you selected **YES** to another primary cancer, please indicate primary cancer:

____

Thank you very much for participating in our study!
