# Supplementary material for: Patient Experiences Regarding Feasibility of Implementing Real-World EQ-5D Collection at an Oncology Centre in Ontario, Canada
Source: Curr Oncol. 2025 May 27;32(6):308. doi: 10.3390/curroncol32060308 (PMC12191542; doi:10.3390/curroncol32060308)
Supplement: Supplementary file 1 [file curroncol-32-00308-s001.zip › Supplementary File S2 Chan_EQ-5D_patient interview guide.pdf]

Study: Real world EQ-5D-5L health utilities to inform cancer drug funding decisions
Principal Investigator: Dr. Kelvin Chan, MD, FRCPC, MSc, PhD

Patient interview guide (1, 2)

Prior to interview:
- Email consent form and screen-shots of questionnaire to participant
- Check that participant signed the consent form

Part 1: Introduction, presenting the project, permission to record interview

Introduction

Welcome and thank you for taking the time to participate in our study. My name is Teresa Tsui. I am a postdoctoral research fellow. I work with Dr. Kelvin Chan at the Sunnybrook Research Institute.

This is [First name, Last name] who is my colleague. [First name] is a [research student/ research coordinator] who will be supporting this interview by taking notes.

Presenting the project

Thank you for completing our EuroQol-5-dimension questionnaire, abbreviated (EQ-5D) questionnaire and for agreeing to provide additional feedback on the questionnaire.

The purpose of this study is to test the feasibility of routinely asking patients to fill in the EQ-5D. We are interested in the feasibility of implementing EQ-5D because we would like to see if we can routinely collect this questionnaire in the future.

[If interview is held towards the beginning of the study]
We recently started collecting EQ-5D responses from patients so we value your input. Based on patient feedback, we plan to make adjustments to our proposed data collection.

OR

[If interview is held towards the end of the study]
We are wrapping up our data collection so this session will help us understand how implementing EQ-5D questionnaires at Sunnybrook has been for patients.

The specific questionnaire you completed is the EQ-5D-5L, which has 5 general dimensions of quality of life and 5 levels within each. The dimensions are: mobility, self-care, usual activities, pain / discomfort, anxiety / depression. Within each dimension, there are 5 levels: for example, no problems, slight problems, moderate problems, severe problems, unable to.

I will refer to the EQ-5D-5L as “EQ-5D”, or “the questionnaire”.

Using EQ-5D quality of life results might help patients and clinicians at the point of care. Additionally, EQ-5D responses can help drug reimbursement agencies understand the effects of cancer drugs on quality of life for patients with cancer.

Today’s interview is to understand your experience of completing the questionnaire.

Your participation in this interview is completely voluntary, and you can stop participating at any time by leaving the session, though you will not be able to withdraw any information you have already provided.

We are committed to creating a safe and respectful space for all participants.

Permission to record the interview and data privacy

This session will be audio recorded and transcribed but your comments will be kept confidential. You will be assigned a participant ID in any transcripts or analyses written after the session.

Do I have your consent to record our interview?

[If patient consents to recording interview, interviewer will turn on recorder.]

[If participant declines being recorded and still wants to participate in interview, interviewer will take notes]

[If recording interview] The recording will be kept secure and will be deleted after it is stored according to Sunnybrook's policy.

There are four general categories of questions in today's interview:

1) Your overall experience with completing the questionnaire
2) How the EQ-5D questionnaire is presented (e.g., tablet, emailed link, paper), and the layout of the questions
3) How often we ask you to complete the EQ-5D questionnaire
4) How we plan to analyze the questionnaire data

Do you have any questions before we get started?

[As patient asks questions, interviewer will answer them]

Part 2. Main Interview

1) Your overall experience with the questionnaire

Would you like to share anything about your experience with the questionnaire?

[If patient requires prompts, the interviewer will give some examples

For example, feel free to mention anything about:
- the questions themselves,
- what was helpful, or challenging about the questions,
- when you were approached to fill in the questions,
- the environment you were in when you filled out the questions.

Please also let us know of any comments or suggestions on what you think we can do differently when we implement the questionnaire more widely.

2) Presenting the EQ-5D questionnaire

a) Mode of administration

When you completed the EQ-5D questionnaire, did you complete it on our tablet or on your own device?

What do you think about completing the questionnaire on our tablet or your own device? What worked well and what would you suggest we can do differently?

Are there other modes of administration you would prefer?

b) Layout of questions

Here are the questions that you completed

[Show patient screen shots of the questions they completed]
[If interview is virtual, interviewer will share screen over Teams and also email patient the handout in advance.]

Feel free to write on (or annotate) the print-out to let us know which aspects of the layout are 1) working well and worth *continuing*, that you suggest we should 2) *start doing differently*, or 3) things to *stop*.

[If interview is held over Teams, offer two options for patient to return annotations via email, or the interviewer can write on the print-out for the patient]

3) How often we ask you to complete the EQ-5D questionnaire

Currently, we ask patients to complete the questionnaire at each chemotherapy visit, for up to four (4) visits.

What are your thoughts on this frequency?

In the future, we hope to make this questionnaire available at all cancer centres in Ontario.

How often do you think is realistic for patients to complete these questionnaires?

4) How we plan to analyze the questionnaire data

As part of my post-doctoral fellowship, I will be analyzing the anonymized questionnaire responses.

Here are some examples of how I plan to analyze the data. Please let me know what you think:

- Factors that are associated with people's different questionnaire responses, such as their clinical characteristics (e.g., type of cancer, stage), timing when they complete the questionnaire, whether they live in a rural or urban area etc.
- I plan to compare the overall EQ-5D responses that we collect from Sunnybrook with those that are published in the literature.

Do you have any ideas on meaningful ways to analyze our EQ-5D health-related quality of life data?

Is there anything that you would like to add to today's interview?

Part 3. Wrap up

Thank you very much for participating in this interview.

We hope that collecting EQ-5D quality of life more widely might help patients and clinicians at the point of care. Additionally, EQ-5D responses can help drug reimbursement agencies understand the effects of cancer drugs on quality of life for patients with cancer.

As a token of our appreciation, we will offer a $50 gift card for your participation. Please check your email for a link that will ask for your email address and preferred e-card. We will arrange to send it to you within the next two weeks.

References

1. Amini M, Oemrawsingh A, Verweij LM, Lingsma HF, Hazelzet JA, Eijkenaar F, et al. Facilitators and barriers for implementing patient-reported outcome measures in clinical care: An academic center's initial experience. Health Policy. 2021;125(9):1247-55.
2. Vennedey V, Hower KI, Hillen H, Ansmann L, Kuntz L, Stock S, et al. Patients' perspectives of facilitators and barriers to patient-centred care: insights from qualitative patient interviews. BMJ Open. 2020;10(5):e033449.
